# Importance of methylammonium iodide partial pressure and evaporation onset for the growth of co-evaporated methylammonium lead iodide absorbers

**DOI:** 10.1038/s41598-021-94689-1

**Published:** 2021-07-27

**Authors:** Karl L. Heinze, Oleksandr Dolynchuk, Thomas Burwig, Jaykumar Vaghani, Roland Scheer, Paul Pistor

**Affiliations:** 1 Thin Film Photovoltaics, Institute of Physics, Martin-Luther-University Halle-Wittenberg, 06120 Halle, Saale Germany; 2 Experimental Polymer Physics, Institute of Physics, Martin-Luther-University Halle-Wittenberg, 06120 Halle, Saale Germany

**Keywords:** Energy science and technology, Engineering, Materials science, Physics, Applied physics, Condensed-matter physics, Electronics, photonics and device physics

## Abstract

Vacuum-based co-evaporation promises to bring perovskite solar cells to larger scales, but details of the film formation from the physical vapor phase are still underexplored. In this work, we investigate the growth of methylammonium lead iodide (*MAPbI*$_3$) absorbers prepared by co-evaporation of methylammonium iodide (*MAI*) and lead iodide (*PbI*$_2$) using an in situ X-ray diffraction setup. This setup allows us to characterize crystallization and phase evolution of the growing thin film. The total chamber pressure strongly increases during *MAI* evaporation. We therefore assume the total chamber pressure to be mainly built up by an *MAI* atmosphere during deposition and use it to control the *MAI* evaporation. At first, we optimize the *MAI* to *PbI*$_2$ impingement ratios by varying the *MAI* pressure at a constant *PbI*$_2$ flux rate. We find a strong dependence of the solar cell device performance on the chamber pressure achieving efficiencies > 14$\%$ in a simple n-i-p structure. On the road to further optimizing the processing conditions we vary the onset time of the *PbI*$_2$ and *MAI* deposition by delaying the start of the *MAI* evaporation by *t* = 0/8/16 min. This way, *PbI*$_2$ nucleates as a seed layer with a thickness of up to approximately 20 nm during this initial stage. Device performance benefits from these *PbI*$_2$ seed layers, which also induce strong preferential thin film orientation as evidenced by grazing incidence wide angle X-ray scattering (GIWAXS) measurements. Our insights into the growth of *MAPbI*$_3$ thin films from the physical vapor phase help to understand the film formation mechanisms and contribute to the further development of *MAPbI*$_3$ and related perovskite absorbers.

## Introduction

In recent years, perovskite solar cells (PSCs), have been subject to intense research due to the outstanding optoelectronic properties of the perovskite absorber^1,2^ and the ease of fabrication through a variety of simple preparation methods^3,4^. Low processing temperatures^5,6^, high compositional versatility^7–11^ and potential usage in cheap, high efficiency single-^12,13^ as well as multi-junction (tandem) solar cells (SCs)^14,15^ have further stimulated research interests.

Methylammonium lead iodide (*MAPbI*$_3$) was the first and has been one of the most investigated materials for perovskite absorbers. Due to their low expense and simplicity in fabrication, up to now most groups have been using wet-chemical deposition approaches such as spin-coating in order to produce *MAPbI*$_3$ layers for structural and optoelectronic analysis as well as photovoltaic applications^16–18^. Although up-scaling of wet-chemical deposition methods is being investigated^3,4^, their large scale preparation is still an open issue in terms of reproducibility, process yield and homogeneity^19^. On the other hand, the historic development of organic light emitting diodes (OLEDs) has shown that physical vapour deposition (PVD) is well suited for thin-film depositions at large scales and has great potential to succeed in the transition from laboratory production to industrial fabrication. Single-junction power conversion efficiencies of above 20$\%$ have already been obtained for co-evaporated *MAPbI*$_3$ PSCs by various groups in a p-i-n configuration^20,21^ and above 16$\%$ in an n-i-p device^20^.

From a variety of different vacuum-based deposition approaches, co-evaporation of the constituent binary halides is arguably the most simple one, and good progress has been made in fabricating efficient devices with this technique. Already in 2013, PVD of $MAPb(I_{1-x}Cl_x)_3$ via dual-source co-evaporation of methylammonium iodide (*MAI*) and $PbCl_2$ showed an advantageous film coverage, an improved layer thickness homogeneity and an increase in SC performance compared to a spin-coated counterpart^22^.

Following works have shown that despite the apparent simplicity of the process, precursor evaporation and the details of the film formation are rather complex. Especially the evaporation and deposition of *MAI* was found to be difficult to control. For example, upon heating, *MAI* evaporates non-directionally and is not withheld by a crucible shutter but diffuses globally into the evaporation chamber. There, it substantially increases the total chamber pressure and is in general not unambigously detected by standard deposition control techniques such as quartz crystal microbalances (QCM)^23^. Therefore, Ono et al. suggested a new approach to monitor the *MAI* evaporation and deposition rate. They relied on using two QCMs, instead of one, where one QCM was facing away from the evaporation sources while the other was facing them directly. With this approach, they managed to verify homogeneous deposition on a 5 $\times$ 5 cm$^2$ sample using X-ray diffraction (XRD)^24^. Later, Liu et al. suggested that *MAI* dissociates during evaporation and is then incorporated into a previously evaporated *PbI*$_2$ layer, introducing the idea that not all *MA*-sites in the perovskite might be occupied by *MA*, but also organic dissociates such as $CH_3$^25^. In 2016, Hsiao et al. showed a two-step approach, depositing *PbI*$_2$ before converting it to perovskite by evaporating *MAI* at chamber pressures ranging from 10$^{-5}$ to 10$^{-3}$ Torr^26^. For a heated chamber and sample they found *MAI* excess as well as deficit were harmful for the performance of their fully evaporated cells^26^. In 2018, Baekbo et al. investigated the *MAI* evaporation behaviour more closely^27^. They installed additional quartz crystal monitors facing away from the evaporation cells and/or with previously evaporated lead halide layers and confirmed earlier results showing a rather low sticking factor for *MAI* and that its deposition was non-directional. Using mass spectrometry they discovered that *MAI* dissociated into mainly two compounds: *CH*$_3$NH$_2$ and *HI*^27^. Borchert et al. found *MAI* impurities to play a significant role in increasing the *MAI* deposition rate, while not playing a role in the SC performance, as long as deposition speed was well controlled^28^. In 2020, Rothmann et al. provided high-resolution scanning transmission electron microscopy (STEM) images of formamidinium lead iodide (*FAPbI*$_3$) as well as *MAPbI*$_3$ absorber layers, revealing the inter-coordinated growth of *PbI*$_2$ and *MAPbI*$_3$ domains. According to this study, a slight excess of *PbI*$_2$ is not harmful for perovskite growth, because it adopts a modified 2H-structure with a seemingly defect-less interface to *MAPbI*$_3$, also not inducing any lattice defects in the *MAPbI*$_3$ crystal^29^.

Even though *MAPbI*$_3$ PVD processing and the non-standard *MAI* sublimation behavior has been intensively investigated in the past, details of the optimal *MAI* processing conditions, such as flux control and optimal flux ratios for co-evaporated absorbers as well as the nature of the film formation remain subject to discussion. More specifically, the impact of the *MAI* flux on the nucleation process, and the general growth path have not been unambigously clarified.

For example, different substrates have been shown to implicate agglomeration of different species at the interfaces. Zhou et al. observed the formation of a thin *PbI*$_2$ layer when depositing it on a single-crystalline *ZnO* (0001) surface via PVD^30^. Olthof et al. detected an organic molecule rich interfacial passivation layer prior to the commencement of the actual crystal growth when depositing *MAPbI*$_3$ via PVD on *MoO*$_3$, Polyethylenimine (*PEIE*), and poly-3,4-ethylendioxithiophene polystyrene sulfonate (*PE-DOT:PSS*) in contrast to a *PbI*$_2$ rich interface layer while depositing on indium tin oxide (*ITO*)^31^. Xu et al. also observed the formation of an interfacial *PbI*$_2$ at the initial growth stage for *ITO*, *PEDOT*/*ITO*, *Si* and glass substrates and found a thin *PbI*$_2$ interlayer to be detrimental for device performance^32^. Contradictory to other publications stating that excess *PbI*$_2$ is beneficial to performance due to a passivation of interfaces and grain boundaries^33,34^, they improved their performance by removing this interlayer and achieved efficiencies of 14.35$\%$^32^.

Another property that has not been investigated thoroughly enough up to now is the influence of crystal orientation on the quality of the perovskite absorber in a SC structure. To the best of our knowledge, attempts to correlate the preferential crystal orientation of *MAPbI*$_3$ absorbers with device performance have only been done for wet-chemical deposition techniques. This said, crystal orientation in polycrystalline perovskite thin films depends strongly on the preparation conditions and is believed to influence electric and electronic properties, as well as improve charge carrier mobility and SC parameters^35,36^. Chen et al. managed to improve the SC performance by implementing a uniform (110) orientation in their *MAPbI*$_3$ absorber compared to a randomly oriented film^37^. At the same time, another investigation has come to the conclusion that orientation plays a minor role compared to defects and impurities in the bulk and at the interface^38^.

Consequently, several open questions remain regarding the optimum film deposition parameters, details of the *MAPbI*$_3$ film formation as well as the influence of orientation on the optoelectronic properties of the absorber. To the best of our knowledge, no attempts have been made to optimize the onset time for the different evaporation components or the thickness of a *PbI*$_2$ seed layer. In this work, we investigate the film formation process under varying processing parameters such as the *MAI* to *PbI*$_2$ evaporation rates for optimized PSCs. We deposit *MAPbI*$_3$ via PVD using dual-source co-evaporation in a self-made vacuum chamber while simultaneously monitoring the crystallization path and phase evolution during deposition in quasi real-time with an in situ XRD (ISXRD) setup attached to the vacuum chamber. Firstly, we adjust the *MAI* pressure in the chamber in three steps, while leaving the *PbI*$_2$ rate constant. The total chamber pressure has been shown to be correlated to the *MAI* evaporation^26,39^ and is assumed to be made up predominantly by the *MAI* partial pressure. In consequence, the *MAI* impingement rate (*MAI* flux towards the substrate surface) was adjusted by controlling the total chamber pressure. Following this line, the chamber pressure was fixed at either 4 $\times$ 10$^{-5}$ mbar, 7.5 $\times$ 10$^{-5}$ mbar or 1.5 $\times$ 10$^{-4}$ mbar. Secondly, in order to deliberately influence the nucleation conditions, the onset (starting) time of the *MAI* and *PbI*$_2$ depositions was systematically varied. This way, a thin *PbI*$_2$ precursor layer was deposited before starting the *MAI* co-deposition. The onset time of *MAI* evaporation was delayed for *t* = 0–16 min with respect to the *PbI*$_2$ onset. A strong correlation of these dynamic processing conditions with the device performance was found. Additionally, with wide angle X-ray scattering (WAXS) we were able to associate the different processing conditions to the growth of perovskite absorbers with rather distinct preferential orientations and relate our findings to the performance of efficient *MAPbI*$_3$ SCs.

## Experimental details

### Sample preparation

For all processes we used 15 ohm/sq indium tin oxide (*ITO*) coated glass substrates (2.5 $\times$ 2.5 cm$^2$), provided by Kintec Company. The substrates were cleaned in 1$\%$ hellmanex solution in de-ionized water, isopropylic alcohol and acetone in an ultrasonic bath for 10 min each. The *ITO* samples were then treated in an ozone plasma for 10 min. Subsequently, 200 $\upmu$l of a 2.6$\%$ colloidal dispersion of tin oxide nanoparticles (*np-SnO*$_2$) was deposited via spin coating at 3000 rpm for 30 s^40^. An additional ozone plasma treatment for 10 min followed before transferring the samples into the vacuum deposition chamber.

### Perovskite deposition

**Figure 1:**
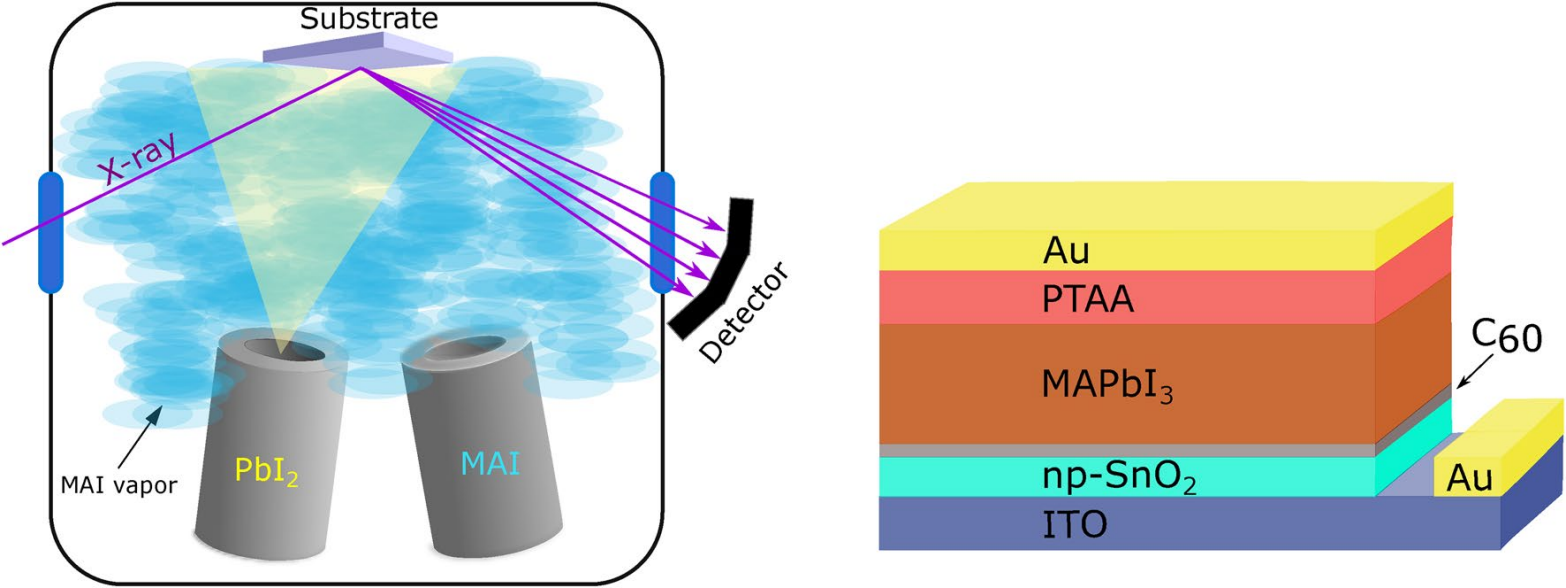
Left: sketch of the vacuum chamber used for the phase analysis with in situ XRD during the deposition of *MAPbI*$_3$ by co-evaporation. The evaporation of *MAI* leads to an overall increase of the global chamber pressure, which in turn was used to control the incorporation of *MAI* into the film. Right: sketch of the solar cell architecture used in this work.

A sketch of the evaporation system with attached in situ X-ray diffraction setup is depicted in Fig. 1, together with a scheme of the device configuration used in this contribution. Base pressure for the start of all processes was between 2 and 2.5 $\times$10$^{-5}$ mbar due to residual leakage through the capton windows that allow the ISXRD measurements to be realized (see below). First, *C*$_{60}$ was evaporated for 5 min at 370 $^\circ$C to form a 10 nm thick buffer layer. Upon cooling of the *C*$_{60}$ crucible, the *PbI*$_2$ and *MAI* crucibles were heated to 288 $^\circ$C and 110/115/125 $^\circ$C, respectively, which resulted in a *PbI*$_2$ flux of 0.2 Å/s, determined via the QCM and scanning electron microscopy (SEM). The *MAI* crucible temperature was then continually adjusted to keep a constant predetermined chamber pressure (either 4 $\times$ 10$^{-5}$ mbar, 7.5 $\times$ 10$^{-5}$ mbar or 1.5 $\times$ 10$^{-4}$ mbar) during the *MAPbI*$_3$ deposition. For this approach, a constant leakage rate and pump capacity is assumed, resulting in a stationary base pressure. The additional chamber pressure increase is then determined by an equilibrium established between evaporation of *MAI* dissociates and the particle drain caused by pumping. The impingement rate of *MAI* dissocates on the substrate therefore directly depends on the chamber pressure under working conditions. The total film thickness was monitored using a quartz crystal microbalance, the total chamber pressure with an Edwards WRGS-NW35 wide range gauge.

### Solar cell completion and measurement

After *MAPbI*$_3$ deposition, the samples were briefly (< 15 min) exposed to air before being sealed in a vacuum-tight bag, and then transferred to a nitrogen filled glovebox within the next 30 min. The hole transport layer Poly(triaryl) amine (*PTAA*) was then prepared by spin coating 100 $\upmu$l of a solution of 6 mg *PTAA* dissolved in 400 $\upmu$l of toluene, to which 3 $\upmu$l of 34 mg/ml lithium bis(trifluoromethanesulfonyl)imide (*Li-TFSI*) in acetonitrile and 3 $\upmu$l of 4-tert-butylpyridine (*4-tBP*) 1:1 in acetonitrile were added. Spin coating took place at 3000 rpm for 30 s. An 80–100 nm thick Gold layer was evaporated in a separate vacuum chamber at 10$^{-5}$ mbar and 2 Å/s.

### Current–voltage characteristics

Current–voltage characteristics were recorded in the dark and under illumination at standard conditions (100 mW/cm$^2$, 25 $^\circ$C) produced by a 300 W Omnilux halogen lamp employing a Keithley 2400 source measure unit. An active area of 0.096 cm$^2$ for the SC measurements was defined by applying appropriate shadow masks.

### Film property measurements

The ISXRD measurements were performed through exchangeable capton windows in the evaporation chamber using Cu-K$_{\alpha }$ radiation with a wavelength of 1.54 Å generated at 1.4 kW (35 kV, 40 mA). Three Dectris Mythen 1 K detector modules are assembled in a row enabling the measurement of 2$\theta$ angles covering a range of 28$^\circ$. The incidence angle was set to 11$^\circ$ resulting in the center of the detector setup (at twice the incidence angle) at a 2$\theta$ angle of 22$^\circ$. This allows an in situ measurement from 8$^\circ$ to 36$^\circ$. Due to the detector assembly (3 modules), there are two blind spots in the diffractograms roughly around 17.3$^\circ$ and 26.7$^\circ$. The K$_{\beta}$ radiation is attenuated through a Ni filter to 5$\%$ of the K$_{\alpha}$ intensity. The $\theta$–2$\theta$ measurements were performed in the same setup right after completion of the evaporation. For the $\theta$–2$\theta$ scans, only the central detector module was used. For more details on the ISXRD setup, please refer to reference^41^. Grazing incidence wide-angle X-ray scattering (GIWAXS) was measured at a pressure of 20–40 $\upmu$bar in a SAXSLAB laboratory setup (Retro-F) (Copenhagen, Denmark) as described elsewhere^42^. The setup used for a reference $\theta$–2$\theta$ scan is described in detail in the supplementary information. SEM was performed with a Zeiss Supra 40 VP.

**Figure 2:**
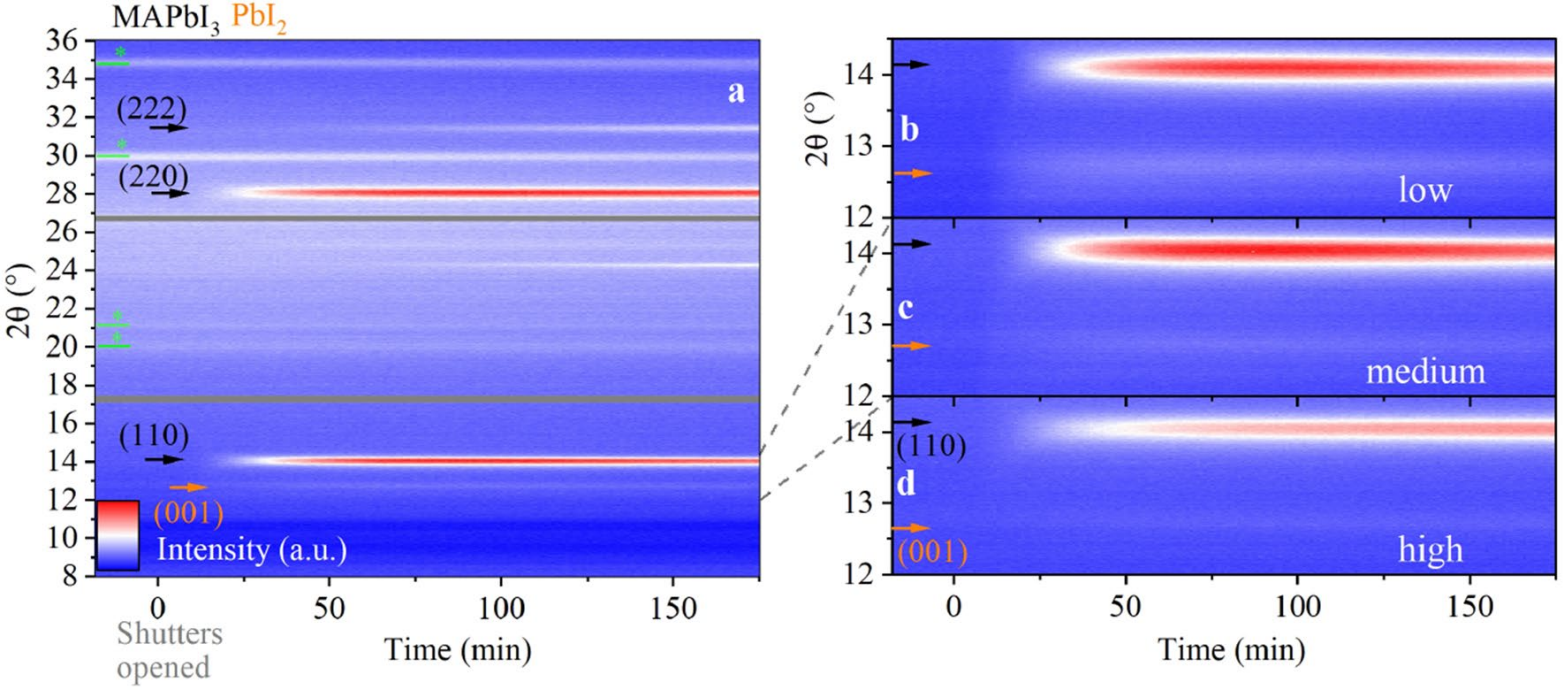
In situ XRD colormaps for vapor deposition *MAPbI*$_3$ at medium (7.5 $\times$ 10$^{-5}$ mbar, **a,c**), low (4 $\times$ 10$^{-5}$ mbar, **b**) and high (1.5 $\times$ 10$^{-4}$ mbar, **d**) pressure. The black arrows indicate the perovskite peaks, while orange and green arrows are used to indicate the *PbI*$_2$ and substrate peaks, respectively.

## Results

Processing conditions were varied and analyzed in view of differences in the perovskite growth and solar cell (SC) performance. At first, the *MAI* to *PbI*$_2$ flux ratio during deposition was varied. For this, the *PbI*$_2$ flux was kept constant, while the *MAI* flux onto the substrate was increased for different deposition runs by controlling the total chamber pressure. Depositions with three different total chamber pressures (low, medium, high) were made and compared. Secondly, using the optimum total chamber pressure, the evaporation onset times of the two components *MAI* and *PbI*$_2$ were varied. The *PbI*$_2$ evaporation onset was set to *t* = 0 while the *MAI* onset time was varied in three steps: *t* = 0/8/16 min. This resulted in *PbI*$_2$ seed layers with different thicknesses prior to the start of the *MAI* deposition.

### *MAI* pressure variation

*MAPbI*$_3$ perovskite layers were deposited at three different total chamber pressures (low: 4 $\times$ 10$^{-5}$ mbar, medium: 7.5 $\times$ 10$^{-5}$ mbar, high: 1.5 $\times$ 10$^{-4}$ mbar) corresponding to three different *MAI* fluxes impinging on the substrate. The growth of the perovskite films was monitored with the ISXRD and the corresponding diffractograms are shown as colormaps in Fig. 2a–d. Here, X-ray intensity is color-coded and the process time evolves from left to right. Figure 2a exemplarily shows the complete evolution of the ISXRD scans for the deposition at medium pressure. The main peaks (e.g. (220) and (110) of *MAPbI*$_3$ can clearly be identified after several minutes of deposition. Figure 2b–d show details of the evolution of the *MAPbI*$_3$ (110) and the *PbI*$_2$ (001) diffraction peaks of the low, medium and high pressure case for comparison. For the high pressure case, the *MAPbI*$_3$ (110) peak intensity is lowest and nearly no *PbI*$_2$ is detected.

**Figure 3:**
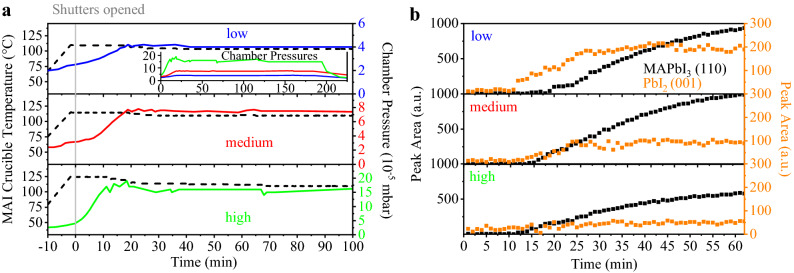
Characterizing initial growth while varying the chamber pressure: (**a**) *MAI* crucible temperature and chamber pressure during the first 100 min of each evaporation process. Inset: chamber pressure plotted for the whole duration of the processes for direct comparison. (**b**) Peak areas for *MAPbI*$_3$ (110) and *PbI*$_2$ (001) peaks for the ISXRD scans for the first 60 min of each evaporation process after opening the shutters.

The *MAI* crucible temperature and the development of the total chamber pressure are illustrated in Fig. 3a. The heatings of the *PbI*$_2$ and *MAI* crucibles started simultaneously. After reaching their respective set temperatures, both shutters were opened and the deposition started (at *t* = 0 min). Once the *MAI* crucible is warmed up, the total chamber pressure rises continuously until reaching the targeted pressure (at *t* = 20 min). In order to keep the total chamber pressure constant, the *MAI* crucible temperature then has to be reduced stepwise. The inset in the top part of Fig. 3a visualizes a direct comparison of the development of the total chamber pressure for the low, medium and high pressure cases.

In Fig. 3b, the evolution of the *MAPbI*$_3$ (110) and *PbI*$_2$ (001) peaks are shown. The peak areas were extracted from the corresponding ISXRD measurements by fitting a quasi-Voigt peak to the respective fixed diffraction angle. Since *MAPbI*$_3$ (110) K$_{\beta }$ and *PbI*$_2$ (001) K$_{\alpha }$ peaks appear at the same angle at 12.7$^\circ$, the K$_{\beta }$ peak of the *MAPbI*$_3$ (110) Bragg reflection had to be considered and substracted for this analysis.

At low pressure (4 $\times$ 10$^{-5}$ mbar), the *PbI*$_2$ peak forms rapidly and grows to a final peak area twice as large as for medium pressure (7.5 $\times$ 10$^{-5}$ mbar) and 4 times higher than for high pressure (1.5 $\times$ 10$^{-4}$ mbar). If we take the integrated intensity to be proportional to the amount of crystalline material in these thin films, this result shows that for the low pressure case, the low *MAI* flux leads to an excess of *PbI*$_2$ forming especially at the beginning of the perovskite deposition.

We specifically find that at low pressure, *PbI*$_2$ starts to form well before *MAPbI*$_3$. This means that for the low pressure case a thin *PbI*$_2$ layer nucleates on the substrate which then acts as a seed layer for the subsequent perovskite growth. In the ISXRD, we observe that the *PbI*$_2$ (001) peak clearly starts to evolve several minutes before the *MAPbI*$_3$ (110) peak. This sequential growth becomes less evident for increasing chamber pressures. In line with the prior argument, a plausible explanation is that the higher *MAI* flux leads to an earlier start of *PbI*$_2$ conversion to *MAPbI*$_3$. As expected, the total peak area of (001) *PbI*$_2$ is also reduced with increased pressure. Interestingly, according to the quartz crystal microbalance measurement, the total deposition rate is reduced with increasing pressure (see supplementary information (SI) Fig. S1) resulting in final thicknesses of 395 nm, 360 nm and 325 nm for low, medium and high pressure, respectively. We explain this by a decrease of the *PbI*$_2$ flux rate due to reduction of the mean free path length caused by an increased number of *MAI* molecules on the way from the crucible to the substrate. Since the *MAI* molecules have a low probability of sticking to the sample if they do not encounter free *PbI*$_2$ to react with^27,28^, a large excess of *MAI* is not expected to lead to an increased growth rate by itself. Following this argument, it comes by no surprise that the perovskite growth as monitored by the ISXRD measurement of the *MAPbI*$_3$ (110) peak is also slowed down at higher pressure.

**Figure 4:**
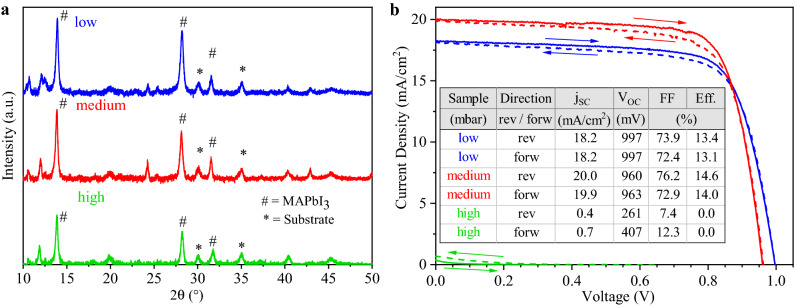
(**a**) XRD scans of the pristine *MAPbI*$_3$ layers prepared at different chamber pressures. (110), (220) and (222) peaks for *MAPbI*$_3$ are indicated by a sharp (#) at 13.9$^\circ$, 28.2$^\circ$ and 31.5$^\circ$, respectively. Substrate peaks are labeled with an asterisk ($*$). (**b**) Current–voltage characteristics for champion cells fabricated at different chamber pressures recorded at 15 mV/s.

In Fig. 4a, $\theta$–2$\theta$ scans of the final films are shown. Sharp (110), (220) and (222) peaks at 13.9$^\circ$, 28.2$^\circ$ and 31.5$^\circ$, respectively, are found, corresponding to the tetragonal room temperature phase of *MAPbI*$_3$. (110) and (220) peak areas are larger when the pressure is reduced, whereas the intensity of the (220) peak remains almost unchanged. This indicates that a more preferential crystallite orientation comes along with increased *PbI*$_2$ contents. The peaks at 12$^\circ$ are interpreted as a setup artifact, since they are already present before the deposition starts. A slight shift in the peak positions as compared to powder references^43^ will be adressed below.

Figure 4b depicts the current–voltage measurements for the respective best cells. The *MAPbI*$_3$ layer prepared at medium pressure performed best, with 14.8$\%$ and 14.0$\%$ measured in reverse and forward voltage sweep direction, respectively, demonstrating the small hysteresis for these devices. Compared to the low pressure preparation, the short circuit current density (j$_{SC}$) is improved from 18.2 to 20.0 mA/cm$^2$, with minor increases also in open circuit voltage (V$_{OC}$) and fill factor (FF). While the preparation at high pressure conditions did show the formation of single phase perovskite material, the absorbers from these conditions did not perform well in devices, with an efficiency of the best cell staying below 0.1$\%$ with nearly no short circuit current. It is assumed that this is due to additional organic phases (such as MAI) forming within the absorber bulk or at the interface to *C*$_{60}$. This leads to the conclusion that the co-evaporation of *MAI* and *PbI*$_2$ is not a simple self-adjusting process, where excess organic species would simply not be incorporated into the perovskite phase, but instead the control of the flux ratios is rather crucial for the formation of high quality SC absorber material. While a lack of *MAI* (excess of *PbI*$_2$) seems to be tolerable to some extent, *MAI* excess is strongly detrimental. This can be seen in the respective SEM images (Fig. S2 of the SI), where the low and medium pressure samples show similar, 100 nm large grains, while high pressure sample exhibits a secondary, organic molecule rich phase.

### *MAI* onset time variation

Above, the medium chamber pressure of 7.5 $\times$ 10$^{-5}$ mbar yielded the highest efficiency devices for a given *PbI*$_2$ flux rate. In the following experiments, this optimized chamber pressure and the *PbI*$_2$ flux rate were kept constant. As we know from previous experiments, the vapor phase surface interaction plays an important role for the nucleation and starting point of the perovskite crystallization, therefore the initial deposition conditions are especially important for the further growth process. In consequence, we analyzed the impact of varied onset times for the *MAI* and *PbI*$_2$ evaporation on *MAPbI*$_3$ growth.

**Figure 5:**
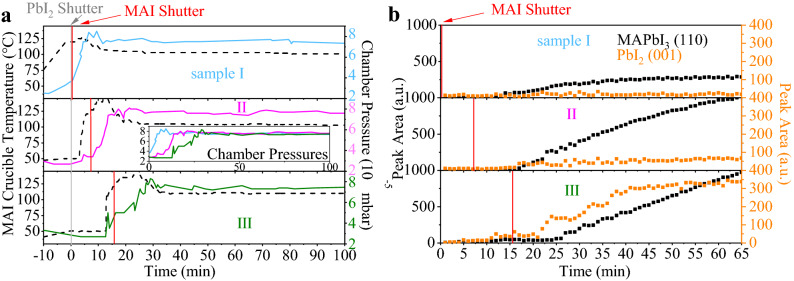
Characterizing initial growth during starting time variation: (**a**) course of the *MAI* crucible temperature (solid lines) and corresponding development of chamber pressure (dashed lines) during the first 100 min for samples I, II and III corresponding to different *MAI* evaporation onset times of *t* = 0/8/16 min. The grey and red lines indicate the evaporation onset times for *PbI*$_2$ and *MAI* shutters. The inset shows a direct comparison of chamber pressures. (**b**) Evolution of (110) *MAPbI*$_3$ (black) and (001) *PbI*$_2$ (orange) peaks for samples I, II and III.

We delayed the starting time of the *MAI* evaporation (crucible temperature ramp up and shutter opening) for several minutes (*t* = 0/8/16 min) with respect to *PbI*$_2$ evaporation onset at *t* = 0 min. These predeposition sequences resulted in pure *PbI*$_2$ precursor layers of 0/10/20 nm thickness (according to the *PbI*$_2$ flux of 0.2 Å/s) that served as seed layers for the subsequent *MAPbI*$_3$ depositions. The nominal chamber pressure was reached at *t* = 5/17/30 min as can be seen in Fig. 5a. For simplicity, the resultant samples will be called samples I, II and III in the following passage.

In this variation, the *MAI* crucible heating ramp was set much faster than in the *MAI* pressure variation, in order to better define the starting time of the *MAI* evaporation. In consequence, the total chamber pressure also builds up faster than in the chamber pressure variation described above. For comparison, the total chamber pressure evolution for medium chamber pressure and best device in the previous section approximately corresponds to sample II in this onset time variation. The $MAPbI_3$ thicknesses were 330 nm, 300 nm and 280 nm for samples I, II and III, respectively.

**Figure 6:**
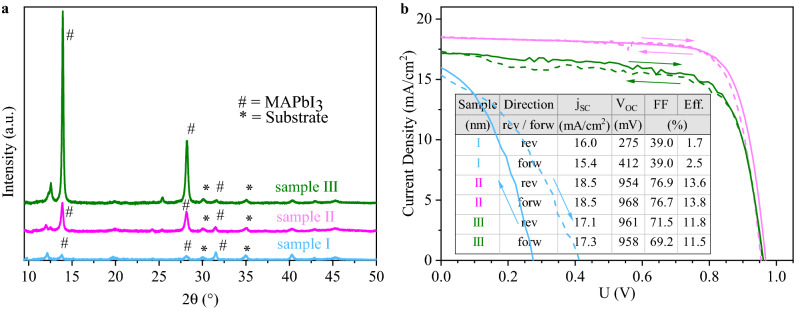
(**a**) $\theta$–2$\theta$ scans of samples I, II and III corresponding to *MAI* evaporation onset times of *t* = 0/8/16 min. (**b**) Current–voltage characteristics for the best *MAPbI*$_3$ cells prepared from samples I, II and III.

Figure 5b depicts the evolution of the *MAPbI*$_3$ (110) and *PbI*$_2$ (001) peaks in our ISXRD scans (see Fig. S3 in the SI). For samples III and II the *PbI*$_2$ (001) peak starts evolving at *t* = 15 min and *t* = 10 min, respectively. It saturates quickly at 50 cps deg for sample II, but increases up to 300 cps deg for sample III, indicating that a predeposited 20 nm pure *PbI*$_2$ layer facilitates further *PbI*$_2$ growth. The *MAPbI*$_3$ (110) peak starts evolving at *t* = 10/15/25 min for samples I, II and III, respectively. Preconditioning of sample III leads to a linear (110) peak growth that does not show any signs of saturation during the time scale considered in Fig. 5b. These peaks can be compared for the finished samples in the $\theta$–2$\theta$ scans in Fig. 6a. For sample I, no *PbI*$_2$ is observed in Fig. 5b. Instead, an early increment of the *MAPbI*$_3$ (110) peak can be seen. This is to be expected, since *MAI* and *PbI*$_2$ fluxes were increased in parallel, leading to an instant conversion of the deposited *PbI*$_2$ to *MAPbI*$_3$.

The strong impact of the *MAI* onset time on the *MAPbI*$_3$ crystal growth can be seen in the $\theta$–2$\theta$ scans displayed in Fig. 6a. By delaying the onset time, a strong increase in the preferential orientation of crystallites in the *MAPbI*$_3$ absorber is observed. The *MAPbI*$_3$ (110) peak area increases sharply from samples I and II to III, indicating a greater proportion of the (110) lattice planes being oriented parallel to the substrate surface. Interestingly, the opposite effect occured for the (222) peak at 31.5$^\circ$. This peak was more pronounced for an earlier onset and was largest for sample I, as expected from the powder diffraction reference with random orientation^43^. This leads to the preliminary conclusion that delaying the *MAI* evaporation onset and consequently depositing a thicker *PbI*$_2$ precursor layer induces *MAPbI*$_3$ crystallite growth with (110) facets orientated in parallel to the substrate surface. Decreasing crystalline domain sizes lead to a peak broadening of the XRD peaks. If the crystallite size was the only origin of peak broadening, the crystallite size would be inversely proportional to the full width at half maximum (FWHM) of the XRD peaks according to the Scherrer equation. While the limited access to the other factors determining the peak broadening in our series prevents a precise quantification of the crystallite size, the FWHM values of the peak fitting analysis presented in Table S1 of the supporting information can still be used for a qualitative discussion. For the series with the delayed *MAI* onset time, we observe a clear decrease of the (110) and (220) FWHM for an increasing onset time, together with the strong increase in peak intensity. In contrast to this, a decrease of the (222) FWHM is observed. This data allows to conclude that the crystallite size along the (110) direction increases with increasing *PbI*$_2$ thickness. Along with the orientational analysis coming up in the next paragraphs, this observation clearly indicates that the *PbI*$_2$ layer not only induces a preferred orientation of the perovskites but also causes its directional growth.

We notice a shift in the diffraction angles compared to a powder diffraction reference provided by Xie et al.^43^. We measured sample I in another setup to account for the shift in our results and note an increase in lattice constant and resulting peak shift to smaller diffraction angles for our sample compared to the reference (see Fig. S4 in the SI).

Current–voltage characteristics of the best devices from the starting time variation are shown in Fig. 6b. Similar to the high pressure case, for sample I very low V$_{OC}$ and FF were measured in both reverse and in forward direction resulting in an average efficiency of 2.1$\%$. The highest efficiencies were achieved for sample II owing to a significantly larger j$_{SC}$ by 1.4 and 1.2 mA/cm$^2$ and FF by 5.4 and 7.5$\%$ for reverse and forward directions, respectively, compared to sample III. This resulted in the sample II best cell’s efficiencies of 13.6 and 13.8$\%$ in reverse and forward directions, respectively.

**Figure 7:**
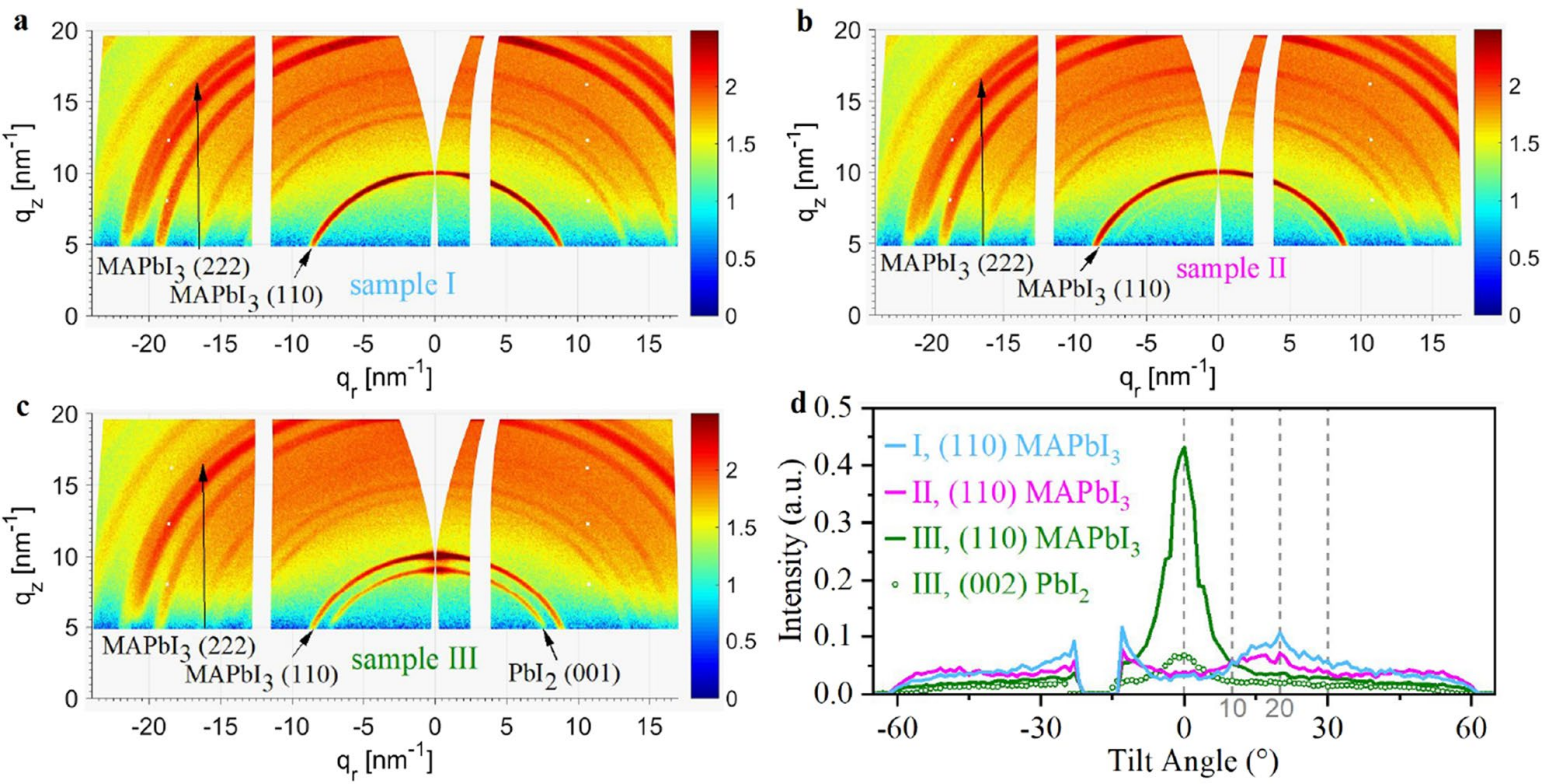
WAXS reciprocal space maps of samples I (**a**), II (**b**) and III (**c**) measured at an incident angle of 6.7$^\circ$. (**d**) The tilt angle dependent peak intensity distribution for the (110) *MAPbI*$_3$ and (002) *PbI*$_2$ diffraction rings. *MAPbI*$_3$ (222) is not fully visible. The tilt angle is defined with respect to the q$_r$-axis as illustrated in Fig. S6 of the supplementary information.

In order to further characterize the distribution of the crystal orientation for different *MAI* evaporation onset times, we also performed wide-angle X-ray scattering (WAXS) measurements. The intensity plots in reciprocal space can be seen in Fig. 7a–c. The images were taken for the same sample sizes and beam parameters and similar layer thicknesses, so it can be assumed that the irradiated volume is similar for all samples. The angle of incidence was 6.7$^\circ$ in order to create a setting close to the Bragg conditions for the *PbI*$_2$ (001) and *MAPbI*$_3$ (110) peaks and fully resolve their intensities along the q$_z$ axis. This is important, as the transformation of the planar detector image into reciprocal space results in blind areas along the q$_z$ axis originating from the Ewald sphere curvature. After transformation, the intensities of *PbI*$_2$ (001) and *MAPbI*$_3$ (110) located on the q$_z$ axis are nearly equivalent to those measured during the $\theta$–2$\theta$ scans presented before (Fig. 6a). Because we chose a high angle of incidence, the sample horizon is located at q$_z$= 5 nm$^{-1}$. Additional WAXS measurements obtained at a small incidence angle of 0.5$^\circ$ confirm that no relevant information on the lattice plain orientation is lost due to the high horizon (see Fig. S5 in the SI). The rings corresponding to the *MAPbI*$_3$ (110) and *PbI*$_2$ (001) reflection are marked with arrows. While the *MAPbI*$_3$ (110) reflection can be clearly identified for all three samples, significant *PbI*$_2$ (001) contributions are only found for sample III. On the *MAPbI*$_3$ (110) ring, the highest intensity is found on the meridian at q$_r$= 0 for sample III, while for samples I and II the maximum intensity is not perpendicular to the sample normal and has an offset of about 20$^\circ$ with respect to the sample normal.

This is further illustrated in Fig. 7d, which shows the intensity distribution of the *MAPbI*$_3$ (110) peak for all three samples under study and one *PbI*$_2$ (001) peak for sample III in dependence of the tilt angle with respect to q$_r$. These data were extracted directly from the detector images instead of the reciprocal graphs, since from there it can be processed directly in the imaging software. As the incident angle of 6.7$^\circ$ almost satisfies the Bragg conditions for the discussed crystal peaks, no angular transformation was applied to these peaks upon conversion of the detector images into the reciprocal space. Therefore, the dependencies in Fig. 7d actually represent the peak intensity distributions in the reciprocal space. The tilt angle is chosen with respect to the sample normal, so that the q$_z$ axis corresponds to a tilt angle of 0$^\circ$ (illustrated in Fig. S6 in the SI). For sample III, it is clearly visible that the *PbI*$_2$ orientation is transferred on to the perovskite (110) orientation. This supports the hypothesis that the *MAPbI*$_3$ grows topotactically on the *PbI*$_2$. For sample II we observe a strong decrease in intensity at 0$^\circ$. Instead, the preferential growth of the (110) plain is tilted by ~20$^\circ$, which is clearly indicated by the broad peak ranging from 10$^\circ$ to 30$^\circ$ and centered at 20$^\circ$. For sample I this effect is even stronger. The same can be assumed at -20$^\circ$, where the detector gap partially masks the intensity distribution. Since the (110) plain is tilted with respect to the q$_z$ axis, another crystal plain is expected to be preferentially oriented along the q$_z$ axis. This is evidenced in Fig. 6a, where the (222) peak intensity was enhanced from sample III to II and I. The angle between the (222) and (110) lattice planes in *MAPbI*$_3$ can be calculated to be 26.4$^\circ$^44^, which lies within the previously indicated tilt angle maximum in the range of 10$^\circ$–30$^\circ$ and therefore confirms this train of thought.

## Discussion

We have investigated different conditions for the *MAI* evaporation in the deposition processes of *MAPbI*$_3$ via co-evaporation. We optimized the processing conditions in terms of device efficiency by controlling the *MAI* flux indirectly via the total chamber pressure. This allowed a more reproducible deposition than other processing parameters such as the *MAI* crucible temperature or control via the quartz microbalance. We observed asymmetries in the influence of an excess of *MAI* and *PbI*$_2$, as an *MAI* excess during deposition (high chamber pressure) or a too early start of *MAI* evaporation completely prevented the deposition of device-grade absorber material, while a mild excess of *PbI*$_2$ was beneficial or tolerable especially at the initial stages of the deposition.

For various vapor deposition techniques, it is assumed that the *MAPbI*$_3$ perovskite phase is grown by intercalation of organic ions into a *PbI*$_2$ structure^45–47^. This growth path is demonstrated by sequential processing, where a *PbI*$_2$ precursor layer is converted into *MAPbI*$_3$ by post-treatment with *MAI* vapor^26,48^. During evaporation of *MAI*, the deposition rate of *MAI* is governed by its vapor partial pressure. During sequential deposition, the *PbI*$_2$ layer is converted top-down. If the *MAI* chamber pressure is too low, this leaves a residual *PbI*$_2$ layer at the bottom^26^. In our experiments, we show a partly sequential (delayed) growth path, which was shown to also result in residual *PbI*$_2$ in the final films, as was observed in the ISXRD. As we decreased the *MAI* impingement rate, the intensity of the *PbI*$_2$ (001) peak in the XRD also increased, pointing towards a reduced conversion to *MAPbI*$_3$. This corresponds well to the *MAI* pressure variation in the sequential deposition process presented by Hsiao et al.^26^.

On multiple occasions, excess *PbI*$_2$ has been shown to improve device performance of *MAPbI*$_3$ solar cells^23,49–51^ by passivating interfacial bonds and bulk defects, as well as improving crystal growth^52^ and reducing hysteresis. In our experiments, remains of minor *PbI*$_2$ secondary phase contributions were detectable for the medium and low pressure processes. A greater amount of *PbI*$_2$ in the bulk, indicated by a *PbI*$_2$ peak in the respective ISXRD scan, could also lead to a passivation of the grain boundaries and was observed to enhance preferential orientation. For the high pressure process, sufficient pressure to convert *PbI*$_2$ to *MAPbI*$_3$ is reached early, leading to full conversion of *PbI*$_2$ and presumably causing an excess of organic species (Fig. S2 in the SI). A high organic content can lead to a fast current-induced degradation of the absorber layer^53^, which would further obstruct charge transport. A resulting organic molecule barrier at the interface^54^ can cause insufficient charge transport and decreasing photo current^46^. The effect of *MAI* excess completely restraining solar cell efficiencies has not been as clear in another study, where major *MAI* excess up to 45% could still yield solar cells with the best efficiencies in that study, although these were distinctly less reproducible than cells with absorbers containing less *MAI*^23^. Our *MAI* evaporation onset delay measurements confirmed that *PbI*$_2$ is especially important as a seed layer at the *C*$_{60}$/*MAPbI*$_3$ interface for producing high efficiency devices. This observation tips the on-going discussion on beneficial/detrimental aspects of a *PbI*$_2$ excess^32,33,52^ towards a positive influence of the latter.

When growing a crystalline layer, its growth path will be decided by a minimization of free energy, whether this is via topotactical growth or surface agglomerates^55,56^. This means that the substrate type and/or the subjacent layer may strongly influence the crystal growth in vapor phase depositions^31,57^. We provide evidence, that in the same way, the crystallite orientation is influenced. In our experiments, depositing *MAPbI*$_3$ on *ITO*/*np-SnO*$_2$/*C*$_{60}$, with no evaporation onset delay for *MAI*, growth of (110) lattice plains tilted by 10$^\circ$ to 30$^\circ$ with respect to the sample normal was induced (Fig. 7). These effects have not been investigated for perovskite absorbers, but play a decisive role for other materials. For example, it has been shown that the interaction of *PMMA* with the substrate is weakened for an increasing layer thickness^58^. For *ZnO*, the optical properties were found to depend on the thickness of a buffer layer^55^. Also, when depositing BaTiO$_3$ the electronic and structural properties were strongly influenced by the thickness of a LaNiO$_3$ buffer layer on a Si substrate^59^. The broad range of preferential growth directions in our perovskite film without *MAI* evaporation onset delay point to different influences from subjacent *ITO*, *np-SnO*$_2$ and *C*$_{60}$ layers, where no dominating effect can be isolated. Delaying the *MAI* onset by 8 min, we observe a shift from wide-spread crystallite orientation towards a slightly preferential growth direction. Further increasing the onset delay, the orientation of the perovskite is almost completely dominated by a *PbI*$_2$ seed layer. The purely inorganic *PbI*$_2$ seed layer is highly oriented itself, likely due to interaction with the substrate. The seed layer screens the substrate from the perovskite and incentivizes a clear crystallite orientation in *MAPbI*$_3$, topotactic with the orientation of the *PbI*$_2$ seed layer.

An approach towards explaining this phenomenon can be taken via the route of different growth paths. For stoichiometric dual-source PVD, *MAPbI*$_3$ follows Volmer–Weber island growth, resulting in randomly oriented small grains with sizes below 100 nm^60^. Typically, Volmer–Weber growth occurs when the cohesion energy between molecules of the deposited material is greater than the adhesion energy between the material and the sample^60^. In this case, agglomerations of the deposited material grow in vertical direction from the sample surface, while the surface-coverage advances slowly. When the adhesion energy is larger, layer-by-layer (Frank–van der Merwe) growth is expected, in which case the considered surface is covered quickly. This could be the case for *PbI*$_2$ seeds in our experiments, since it grows in a highly oriented manner. It has often been shown, that lead halides improve the sticking factor for *MAI*^27,28^, and *MAI* can intercalate into the *PbI*$_2$ lattice to form *MAPbI*$_3$^26,61^. This hints towards an increase in adhesion energy for *MAI* on the substrate covered with *PbI*$_2$. In consequence, a transition to layer-by-layer growth for the perovskite could take place, thus inducing the observed preferential orientation.

The orientation is suspected to influence the fundamental properties of perovskites^35^. However, it is expected to play a minor role for the optical properties of *MAPbI*$_3$, and the correlations between electronic properties and preferential orientation have not yet been clarified^38^. Dedicated to this question, Chen et al. studied the wet-chemical post-deposition of *MACl* on spin-coated *MAPbI*$_3$^37^. By the post-deposition procedure, the grain size was increased and preferential orientation of the perovskite crystallites improved, resulting in a reproducible increase in SC efficiencies from average values of 11–15$\%$. Since the $V_{OC}$ was not improved, the increase in efficiency was not attributed to larger crystallite sizes or a possible trap site passiviation by *Cl*. It was rather attributed to the improved orientation and a corresponding decrease in series resistance^37^. From our SCs’ performances it cannot be deduced, whether changing orientation plays a role for charge transport and SC performance. On the one side, the strong impact of the *PbI*$_2$ seed layer on preferential orientation of *MAPbI*$_3$ was shown. On the other side, the importance of the seed layer for SC performance could be seen, but without direct proof that this is related to film orientation, as it could also be caused for example by a passivating effect of the residual *PbI*$_2$ layer. The thickest *PbI*$_2$ layer at latest *MAI* evaporation onset could improve charge transport in the bulk while, due to its low conductivity, also acting as a barrier at the *C*$_{60}$/*MAPbI*$_3$ interface. Further focused investigation observing the coupling of the preferential crystallite growth and SC performance are suggested in the future.

## Conclusion

In this study, we introduced the chamber pressure as a parameter for controlling the methylammonium iodide (*MAI*) evaporation and respective impingement rate during the growth of methylammonium lead iodide (*MAPbI*$_3$) absorbers for perovskite solar cells. We observed a strong influence of chamber pressure on the absorber film formation and, consequently, on cell performance. At a given *PbI*$_2$ flux of 0.2 Å/*s*, we found an optimum chamber pressure for *MAI* deposition at 7.5 $\times$ 10$^{-5}$ mbar. Increasing the chamber pressure further up to 1.5 $\times$ 10$^{-4}$ mbar was strongly detrimental to the functionality of the absorber, presumably because of an excess of organic species in the bulk and/or at the electron transport layer interface. At optimum chamber pressure, a small amount of excess *PbI*$_2$ was found and efficiencies above 14$\%$ were achieved with low hysteresis.

Using a specially designed in situ setup, we were able to investigate the initial absorber growth for the first time using X-ray diffraction (XRD). At optimum chamber pressure, we noticed that initially a *PbI*$_2$ layer is deposited, which then acted as a seed for perovskite growth. This observation is in accordance with the slow rise in chamber pressure, which was characteristic for the evaporation of *MAI*.

A controlled deposition of *PbI*$_2$ seed layers showed that *PbI*$_2$ has a strong influence on the crystallization and growth behaviour of the perovskite. Without *PbI*$_2$ seed layer, low intensity XRD peaks were observed and the prepared solar cells showed efficiencies below 3$\%$. Delaying the *MAI* onset time by 8–16 min. drastically enhanced XRD peak intensities and led to efficient solar cells.

This work based on ISXRD provides a detailed characterization of the thin film growth using a new pressure-reliant approach for the deposition of *MAPbI*$_3$ perovskite absorbers. We provide further evidence that *PbI*$_2$ plays a paramount role at the interface and for the initial growth of the perovskite, vastly determining also the bulk of the absorber in perovskite solar cells. Consequently, we show that the use of thin *PbI*$_2$ seed layers enables the growth of highly crystalline and high quality organic–inorganic perovskites with physical vapor deposition techniques, which opens new optimization pathways and process developments for the deposition of perovskite thin films from the physical vapor phase.

## Supplementary Information

Supplementary Information 1.
