# Supplementary material for: Importance of methylammonium iodide partial pressure and evaporation onset for the growth of co-evaporated methylammonium lead iodide absorbers
Source: Sci Rep. 2021 Jul 27;11:15299. doi: 10.1038/s41598-021-94689-1 (PMC8316399; doi:10.1038/s41598-021-94689-1)
Supplement: Supplementary file 1 — Supplementary Information 1. [file 41598_2021_94689_MOESM1_ESM.pdf]

Supporting Information: Importance of Methylammonium Iodide Partial Pressure and Evaporation Onset for the Growth of Co-Evaporated Methylammonium Lead Iodide Absorbers

Karl L. Heinze[1], Oleksandr Dolynchuk[2], Thomas Burwig[1], Jaykumar Vaghani[1], Roland Scheer[1], and Paul Pistor[1,*]

[1]Thin Film Photovoltaics, Institute of Physics, Martin-Luther-University Halle-Wittenberg, Halle (Saale) 06120, Germany
[2]Experimental Polymer Physics, Institute of Physics, Martin-Luther-University Halle-Wittenberg, Halle (Saale) 06120, Germany
[*]paul.pistor@physik.uni-halle.de

The following figures are referenced in the main manuscript and support the understanding of the experimental proceedings and results. In the *MAI* flux variation, total chamber pressures are named low, medium and high. These correspond to $4\cdot10^{-5}$ mbar, $7.5\cdot10^{-5}$ mbar and $1.5\cdot10^{-4}$ mbar, respectively. In the *MAI* evaporation onset variation, samples I, II and III correspond to onset times of 0 / 8 / 16 min, respectively. In this time, PbI_2 seed layers with thicknesses of 0 / 10 / 20 nm are grown, according to the PbI_2 flux of 0.2 Å/s.
Fig. S1 shows the deposition rate of $MAPbI_3$ at different chamber pressures measured by the quartz crystal monitor. Even though we would expect a higher total deposition rate of *MAI* at higher chamber pressures, an increase in chamber pressure resulted in slower film growth, by means of this measurement. A limiting factor for deposition can be the mean free path length for PbI_2, which is strongly influenced by the chamber pressure. When less PbI_2 is deposited, the sticking factor of *MAI* is also greatly reduced[1], resulting in an overall slower film growth.
In Fig. S2 the morphology of $MAPbI_3$ absorbers prepared at varying chamber pressures on $ITO/np\text{-}SnO_2/C_{60}$ is compared. The smallest crystals were observed for the lowest pressure, increasing in size for increased pressure. In the high pressure process, a phase segregation is visible. The new phase appears to consist of larger crystallites and is very volatile under electron beam exposure. This is most likely due to a high organic content[2,3].
Fig. S3 depicts the *in situ* XRD colormaps of samples I, II and III of the *MAI* evaporation onset variation. A distinguishing feature is the $MAPbI_3$ (110) peak intensity, which is strongly enhanced for an increase in *MAI* onset time. PbI_2 peaks are visible for samples II and III.
Fig. S4 depicts an XRD scan for sample I recorded in another setup than the setup that was used for *in situ* XRD and XRD measurements in the main text. This was done to check for a possible peak shift in the measurements in the main text in comparison to literature.
In fig. S5, grazing incidence wide angle X-ray scattering (GIWAXS) measurements recorded at 0.5° for samples I, II and III are shown. The angle of incidence of 0.5° visualizes effects below the horizon of q_z= 5 nm^{-1}, which could not be seen in the GIWAXS measurements in the main text recorded at an angle of incidence of 6.7°. The broadening of the peak rings observed here, compared to an angle of incidence of 6.7°, results from the large illuminated area at this low angle of incidence. Resolution of the reciprocal space map is diminished around the q_z axis for higher values of q_z due to the Ewald sphere curvature.
Fig. S6 illustrates chosen geometry to define the tilt angle with respect to the sample surface.

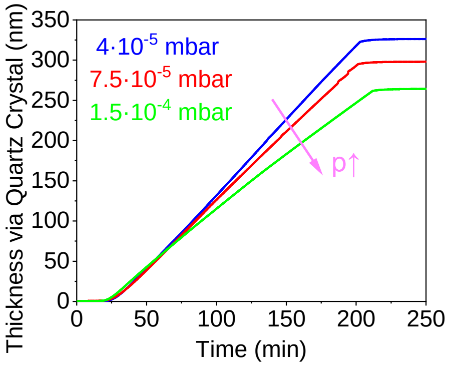

Figure S1. Varying perovskite thicknesses for different chamber pressures measured via the quartz crystal monitor.

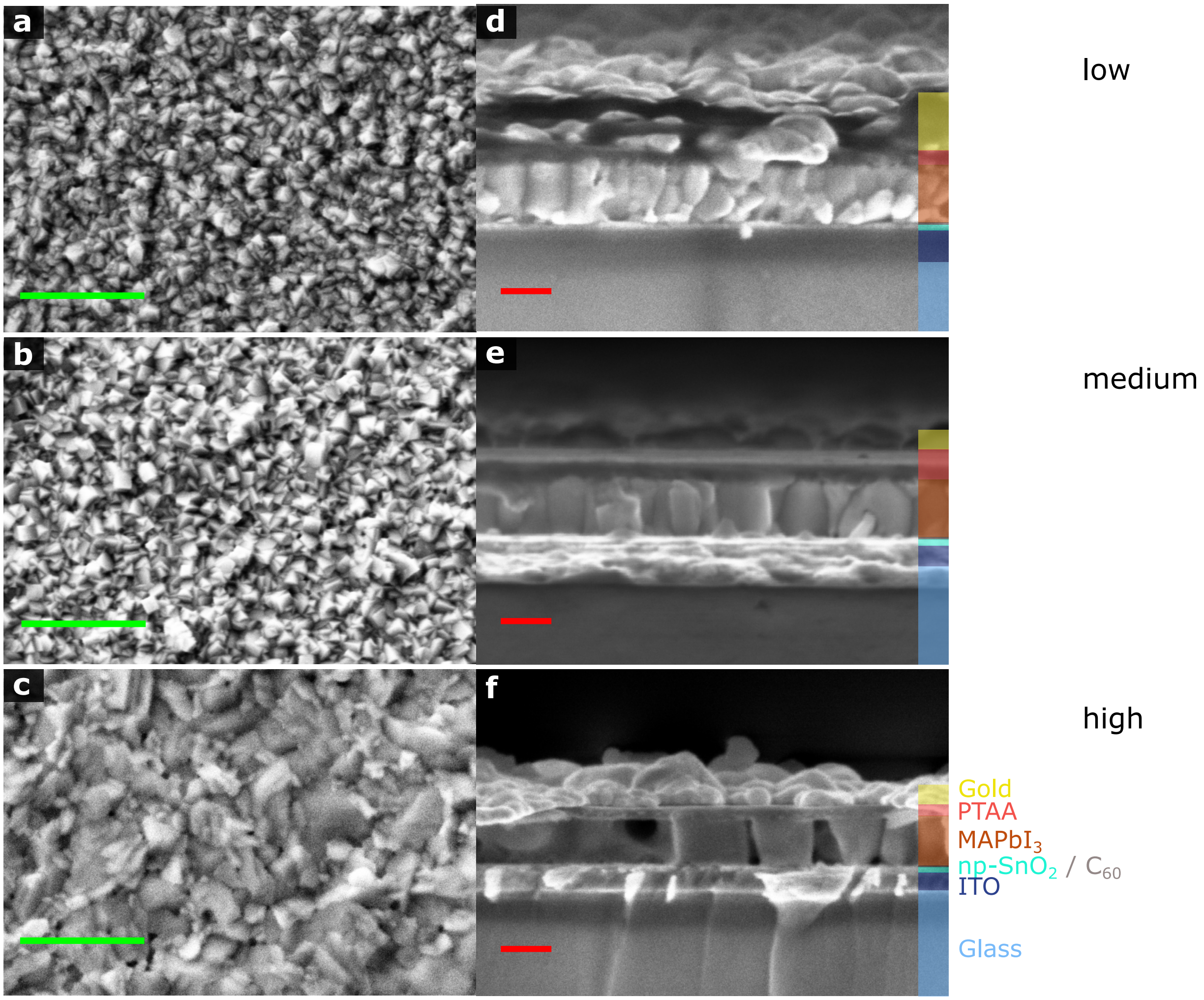

Figure S2. Left: SEM top-view images of the pristine perovskite layers prepared at low (**a**), medium (**b**) and high (**c**) chamber pressures. The green scale-bar represents 1 μm. Right: Corresponding cross-sectional images of the finished solar cells for low (**d**), medium (**e**) and high (**f**) pressures. The red scale-bar represents 200 nm.

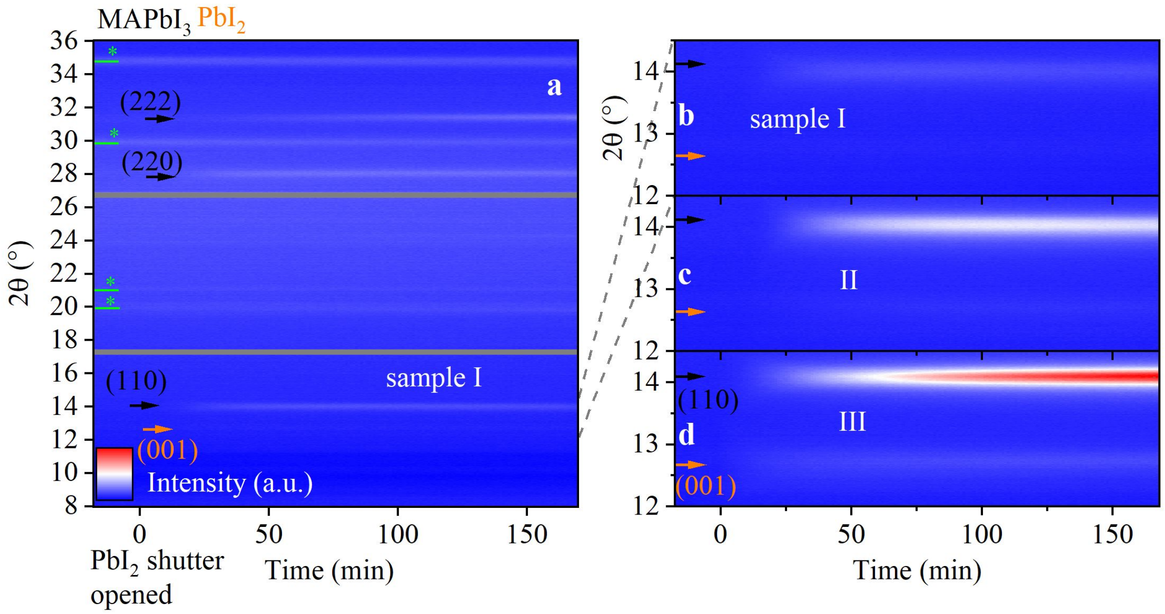

Figure S3. *In situ* XRD diagrams for samples I (**a**, **b**), II (**c**) and III (**d**) corresponding to *MAI* evaporation onset times of $t = 0$ / 8 / 16 min. The black arrows indicate the perovskite peaks, while orange and green arrows are used to indicate the PbI_2 and substrate peaks, respectively.

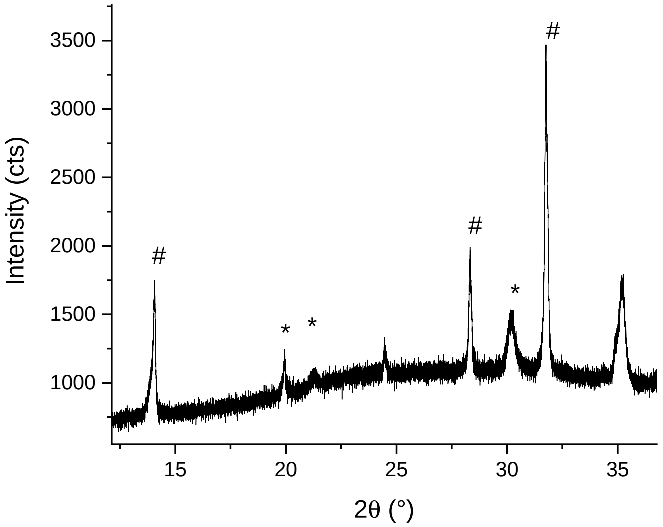

Figure S4. XRD scan for sample I retaken in another setup to determine shifts in the diffraction peaks. ♯ from left to right mark (110), (220) peaks and (222)/(310) double peak. ∗ label substrate peaks.

Measurement The reference XRD scan was measured on a PANalytical Empyrean x-ray diffractometer (Amelo, Netherlands). The incoming Cu-Kα (λ = 0.15418 nm) radiation passed through a 0.04 rad Soller slit, a 10 mm mask, and a programmable divergence slit before interacting with the sample. The irradiated length on the sample's surface was 10 mm. The diffracted X-ray radiation passed through a programmable anti-scatter slit, a 0.04 rad Soller slit, and a 0.02 mm thick nickel beta-filter used to filter out the Cu-Kβ radiation. A PIXcel3D detector (Medipix2, Meyrin, Switzerland) was used to collect the diffracted radiation. Operating parameters of the X-ray tube were at 40 kV and 40 mA.

Table S1. Integrated area (A) and full width at half maximum (FWHM) from the peak analysis of the $MAPbI_3$ (110), (220) and (222) diffraction peaks taken from the θ-2θ scans shown in the main document for different total chamber pressures (figure 4 a) and for different MAI onset times (figure 6 a). Peak properties were determined by fitting with a Lorentz function.

Pressure	A (110) [ctsdeg]	FWHM (110) [cts]	A (220) [ctsdeg]	FWHM (220) [cts]	A (222) [ctsdeg]	FWHM (222) [cts]
low	1271	0.28	1510	0.37	285	0.29
medium	1124	0.26	933	0.33	306	0.24
high	870	0.27	633	0.31	340	0.40

MAI onset [min]	A (110) [ctsdeg]	FWHM (110) [cts]	A (220) [ctsdeg]	FWHM (220) [cts]	A (222) [ctsdeg]	FWHM (222) [cts]
0	316	0.34	319	0.42	432	0.26
8	1903	0.28	1368	0.33	239	0.29
16	8556	0.18	3754	0.27	163	0.33

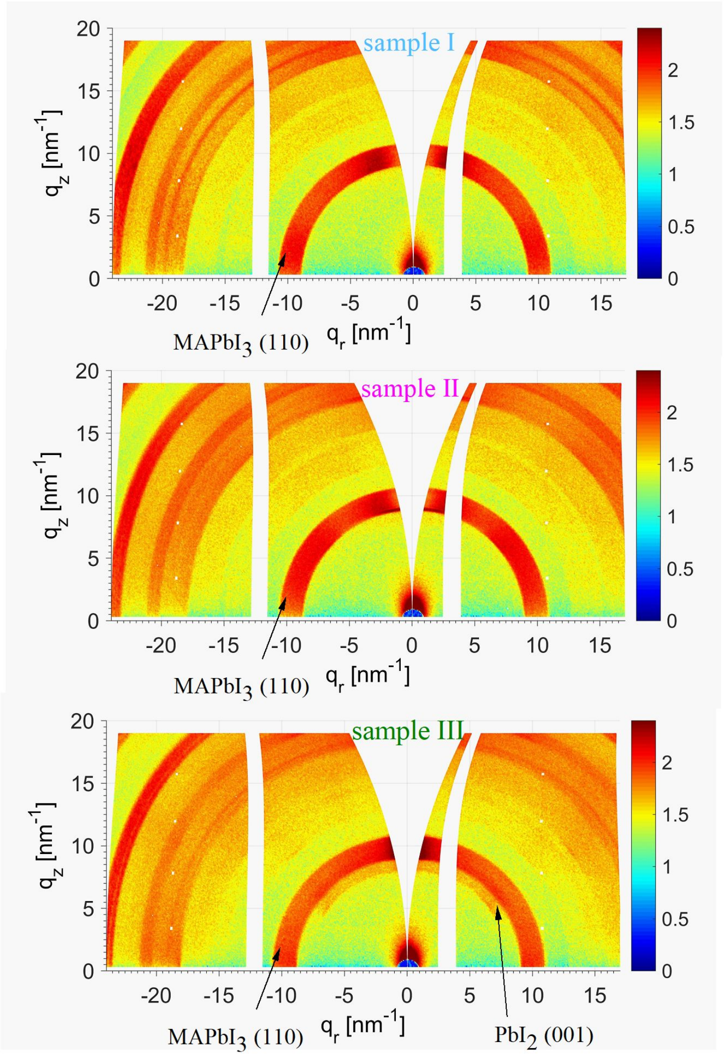

Figure S5. GIWAXS measurements at an incident angle of 0.5° of samples I, II and III corresponding to *MAI* evaporation onset times of $t = 0 / 8 / 16$ min.

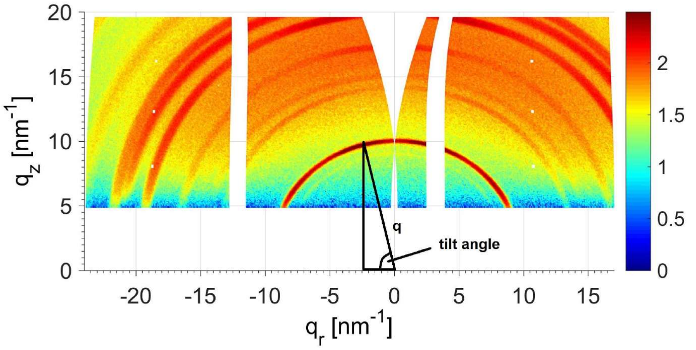

Figure S6. Sketch to clarify the definition of the tilt angle used in fig. **6d**.

References

1. Kim, B.-S., Gil-Escrig, L., Sessolo, M. & Bolink, H. J. Deposition Kinetics and Compositional Control of Vacuum-Processed CH3NH3PbI3 Perovskite. *The J. Phys. Chem. Lett.* **11**, 6852–6859, DOI: 10.1021/acs.jpclett.0c01995 (2020). Publisher: American Chemical Society.
2. Rothmann, M. U. *et al.* Structural and Chemical Changes to CH3NH3PbI3 Induced by Electron and Gallium Ion Beams. *Adv. Mater.* **30**, 1800629, DOI: https://doi.org/10.1002/adma.201800629 (2018). _eprint: https://onlinelibrary.wiley.com/doi/pdf/10.1002/adma.201800629.
3. Ran, J. *et al.* Electron-Beam-Related Studies of Halide Perovskites: Challenges and Opportunities. *Adv. Energy Mater.* **10**, 1903191, DOI: https://doi.org/10.1002/aenm.201903191 (2020). _eprint: https://onlinelibrary.wiley.com/doi/pdf/10.1002/aenm.201903191.
